# ^18^F−Prostate−Specific Membrane Antigen PET/CT imaging for potentially resectable pancreatic cancer (PANSCAN−2): a phase I/II study

**DOI:** 10.1186/s40644-025-00822-y

**Published:** 2025-01-14

**Authors:** Jisce R. Puik, Thomas T. Poels, Gerrit K. J. Hooijer, Matthijs C. F. Cysouw, Joanne Verheij, Johanna W. Wilmink, Elisa Giovannetti, Geert Kazemier, Arantza Farina Sarasqueta, Daniela E. Oprea-Lager, Rutger-Jan Swijnenburg

**Affiliations:** 1 Department of Surgery, Amsterdam UMC Location Vrije Universiteit Amsterdam, De Boelelaan 1117, Amsterdam, the Netherlands; 2 Cancer Center Amsterdam, Imaging and Biomarkers, Amsterdam, The Netherlands; 3 Department of Pathology, UMC Location University of Amsterdam, Meibergdreef 9, Amsterdam, the Netherlands; 4 Department of Radiology and Nuclear Medicine, Amsterdam UMC Location Vrije Universiteit Amsterdam, De Boelelaan 1117, Amsterdam, the Netherlands; 5 Department of Medical Oncology, Amsterdam UMC Location Vrije Universiteit Amsterdam, De Boelelaan 1117, Amsterdam, the Netherlands; 6 Cancer Pharmacology Lab, Fondazione Pisana Per La Scienza, Pisa, Italy; 7 Department of Surgery, Amsterdam UMC Location University of Amsterdam, Meibergdreef 9, Amsterdam, the Netherlands

**Keywords:** PSMA, PET/CT, PDAC, Pancreatic cancer, Diagnosis

## Abstract

**Background:**

Current diagnostic imaging modalities have limited ability to differentiate between malignant and benign pancreaticobiliary disease, and lack accuracy in detecting lymph node metastases. ^18^F-Prostate-Specific Membrane Antigen (PSMA) PET/CT is an imaging modality used for staging of prostate cancer, but has incidentally also identified PSMA-avid pancreatic lesions, histologically characterized as pancreatic ductal adenocarcinoma (PDAC). This phase I/II study aimed to assess the feasibility of ^18^F-PSMA PET/CT to detect PDAC.

**Methods:**

Seventeen patients with clinically resectable PDAC underwent ^18^F-PSMA PET/CT prior to surgical resection. Images were analyzed both visually and (semi)quantitatively by deriving the maximum standardized uptake value (SUV_max_) and tumor-to-background ratio (TBR). TBR was defined as the ratio between SUV_max_ of the primary tumor divided by SUV_max_ of the aortic blood pool. Finally, tracer uptake on PET was correlated to tissue expression of PSMA in surgical specimens.

**Results:**

Out of 17 PSMA PET/CT scans, 13 scans demonstrated positive PSMA tracer uptake, with a mean SUV_max_ of 5.0 ± 1.3. The suspected primary tumor was detectable (TBR ≥ 2) with a mean TBR of 3.3 ± 1.3. For histologically confirmed PDAC, mean SUV_max_ and mean TBR were 4.9 ± 1.2 and 3.3 ± 1.5, respectively. Although eight patients had histologically confirmed regional lymph node metastases and two patients had distant metastases, none of these metastases demonstrated ^18^F-PSMA uptake. There was no correlation between ^18^F-PSMA PET/CT SUV_max_ and tissue expression of PSMA in surgical specimens.

**Conclusions:**

^18^F-PSMA PET/CT was able to detect several pancreaticobiliary cancers, including PDAC. However, uptake was generally low, not specific to PDAC and no tracer uptake was observed in lymph node or distant metastases. The added value of PSMA PET in this setting appears to be limited.

**Trial registration:**

The trial is registered as PANSCAN-2 in the European Clinical Trials Database (EudraCT number: 2020–002185-14).

**Graphical Abstract:**

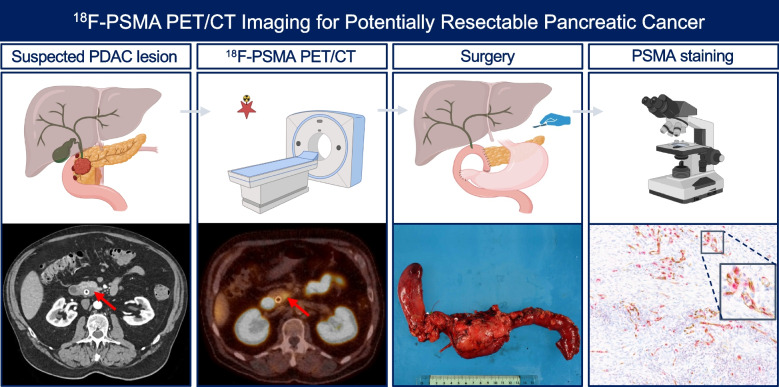

**Supplementary Information:**

The online version contains supplementary material available at 10.1186/s40644-025-00822-y.

## Background

Pancreatic ductal adenocarcinoma (PDAC) is notorious for its aggressive nature and poor prognosis, with a 5-year survival rate of approximately 12% [1, 2]. One of the major challenges is accurate diagnosis and staging, as several benign and malignant pancreaticobiliary diseases strongly mimic PDAC [3]. To characterize pancreaticobiliary lesions, assess resectability, and determine the preoperative lymph node status, patients often undergo computed tomography (CT), endoscopic retrograde cholangiopancreatography (ERCP) with brush cytology, and endoscopic ultrasound guided fine-needle biopsy (EUS-FNB) [4]. However, these tests lack sufficient diagnostic accuracy, oftentimes necessitating repeated (invasive) procedures [5–7]. Indeed, 10% of the patients that undergo surgical resection for suspected PDAC is finally diagnosed with benign disease [8]. Moreover, almost 50–60% of the patients with PDAC that undergo curative-intent surgery show early recurrence within 12 months [8, 9]. Undetectable metastases during the diagnostic process may contribute to early metastatic recurrence after upfront surgical resection [9, 10].

Currently, ^18^F-fluorodeoxyglucose (FDG) PET/CT may be offered to patients for staging and evaluation of possible extra-pancreatic metastases, or to patients undergoing neoadjuvant or induction therapy [4, 11, 12]. However, routine use of PET/CT in the diagnostic workup of patients with PDAC is still variable, especially due to non-specific FDG-uptake in benign inflammatory and other malignant pancreaticobiliary disease [4, 12, 13]. Moreover, these disease entities are typically characterized by extensive desmoplastic reaction, which makes differential diagnosis of PDAC challenging and can contribute to a lower intensity of PDAC on ^18^F-FDG PET/CT [14]. Additionally, elevated glucose levels in hyperglycemic patients may interfere with the level of ^18^F-FDG uptake [13, 15]. Molecular imaging with more tumor-specific radiotracers may have the potential to overcome these issues and improve diagnostic accuracy.

Recently, several case reports have published on incidental findings of histologically confirmed PDAC in patients undergoing prostate-specific membrane antigen (PSMA-) targeted PET imaging for the staging of prostate cancer [16–19]. PSMA PET/CT targets the transmembrane glycoprotein PSMA, which is aberrantly expressed in the cell membrane of epithelial prostate cancer cells [20–23]. However, PSMA is not specific to prostate cancer. In tissue analysis of PDAC, PSMA appears to be aberrantly expressed in the endothelium of tumor-associated neovasculature [24–26]. Given these findings, ^18^F-PSMA PET/CT may be of diagnostic value for patients with PDAC. This prospective phase I/II study assessed the diagnostic applicability of ^18^F-PSMA PET/CT in patients with suspected, potentially resectable PDAC.

## Methods

### Study design

The complete version of the Methods is provided in Supplemental File 1. The PANSCAN-2 study was a prospective phase I/II study that aimed to determine whether PDAC can be detected and staged by ^18^F-PMSA PET/CT imaging. Additionally, we aimed to assess the relation between ^18^F-PSMA tracer signal and PSMA protein expression on tissue samples by immunohistochemical (IHC) analysis. The protocol was approved by the local Medical Ethical Board of the Amsterdam UMC (NL73356.029.20), and has been registered as PANSCAN-2 in the European Clinical Trials Database (EudraCT number: 2020–002185-14) [27]. Written informed consent was obtained from each patient.

Patients were included, if they presented with clinically suspected PDAC and were eligible for upfront curative-intent surgery. In addition to standard-of-care diagnostic workup [4], patients underwent an ^18^F-PSMA PET/low-dose CT scan prior to surgery. Scans were scored both visually and (semi)quantitatively. Following surgical resection, formalin-fixed paraffin-embedded (FFPE) tumor tissue slides were stained and scored for PSMA expression. This study aimed to include a total of fifteen patients that had undergone surgical resection. No sample size calculations were performed because of the exploratory character of this pilot study.

### ^18^F-PSMA PET/CT acquisition and analysis

The investigational radiopharmaceutical used was ^18^F-DCFPyL [28]. Two hours after intravenous injection of 300 MBq (± 10%) ^18^F-DCFPyL, a static ^18^F-PSMA PET/low-dose CT (120–140 kV, 40–80 mAs) was acquired from the skull base to the mid-thigh using a PET/CT scanner equipped with time-of-flight (TOF) technology (Philips Healthcare, Best, the Netherlands). Low-dose CT scans were used for anatomic correlation and attenuation correction. Scans were reviewed by an experienced nuclear medicine physician in accordance with European Association of Nuclear Medicine (EANM) E-PSMA standardized reporting guidelines v1.0 [29]. When PSMA expression visually exceeded the blood pool uptake, a lesion was reported as ‘positive’. Positive lesions were then scored using the EANM 4-point grading scale that relates to visual score, as described in Additional File 1 [29]. For (semi)quantitative analysis, PSMA uptake was derived using maximum standardized uptake value (SUV_max_) normalized to body weight. Tumor-to-background ratio (TBR) was defined as the ratio between SUV_max_ of the primary tumor divided by SUV_max_ of the aortic blood pool.

### Immunohistochemistry

A representative FFPE tissue block was selected for the presence of tumor tissue. Sections of 4 µm were obtained. A double staining was performed with PSMA antibody (Clone 3E6, 1:100, DAKO) and Ets Related Gene (ERG; clone EP111, 1:200, Cell Marque Tissue Diagnostics). PSMA expression was determined as the ratio positive vessels in relation to ERG positive vessels in the tumor bed. Additionally, staining intensity was determined as weak, moderate or strong.

### Statistical analysis

Statistical analyses were performed using SPSS version 28 (IBM SPSS). Data is displayed as mean ± standard deviation. Correlation analysis between SUV_max_ and the PSMA/ERG ratio was performed using logistic regression analysis. A *p*-value of < 0.05 was considered statistically significant.

## Results

### Patient characteristics

Between February 2021 and June 2023, seventeen consecutive patients were prospectively enrolled at the Amsterdam UMC, location VUmc. Patient characteristics, PSMA PET/CT scores and tissue PSMA expression results are summarized in Table 1. Two patients did not undergo surgery due to histologically confirmed metastatic disease, detected on a preoperatively repeated CT or during diagnostic laparoscopy. Therefore, two additional patients were included, resulting in a total cohort of seventeen patients of whom fifteen had undergone surgical resection. Furthermore, diagnostic CT imaging showed a second primary malignancy in two patients: one patient was diagnosed with a concomitant primary gastric signet ring cell carcinoma and one patient with a renal cell carcinoma. Although all pancreatic lesions were suspected PDAC, histopathological analysis following surgery demonstrated several diagnoses: PDAC (*n* = 8), adenocarcinoma of pancreaticobiliary origin (*n* = 2), cholangiocarcinoma (*n* = 1), duodenal carcinoma (*n* = 1), PDAC associated with intraductal papillary mucinous neoplasm (IPMN; *n* = 1), an undifferentiated carcinoma (*n* = 1) and non-invasive IPMN (*n* = 1).

**Table 1:** Patient characteristics and PSMA expression

N	Age	Sex	Tumor location	Histology	Origin	pTNM stage^a^	Locoregional lymph nodes^b^	Tumor differentiation	DM (mm)^c^	PSMA uptake^d^	Visual score^e^	SUVmax	TBR	PSMA + /ERG + (%)	Staining vessels	Acinar parenchyma
1	71	F	head	ductal adenocarcinoma	uncertain	T2N2	3/8	intermediate	28	yes	3	4.48	2.91	30–40	+	-
2	66	F	body-tail	ductal adenocarcinoma	pancreas	T2N1	2/9	intermediate	33	yes	2	5.40	2.38	20–30	+	-
3	68	F	juxta-papillary	carcinoma	uncertain	n.a	0/22	undifferentiated	41	yes	2–3	7.07	2.24	60	+	-
4	70	M	uncinate process	liver biopsy: adenocarcinoma	likely pancreas	TxNxM1	unknown	unknown	18	yes	3	6.83	3.69	10–12	+	n.a
5	66	F	pancreaticoduodenal groove	adenocarcinoma	duodenum	T3N0	0/16	intermediate	65	yes	1–2	4.00	2.56	30–40	+	-
6	76	F	head	ductal adenocarcinoma	pancreas	T1bN0	0/24	well	16	yes	2–3	6.72	3.63	10	+ +	-
7	74	M	head	adenocarcinoma in IPMN	pancreas	T1cN2	7/16	well	19	no	0	n.a	n.a	10–20	+	-
8	73	F	head	ductal adenocarcinoma	pancreas	T1cN0	0/19	intermediate	14	yes	3	5.43	4.18	40	+	-
9	70	M	uncinate process	non-invasive IPMN, intestinal type	pancreas	n.a	0/28	n.a	n.a	yes	1	5.07	5.28	10	+	-
10	66	F	head	ductal adenocarcinoma	pancreas	T1cN1	1/13	intermediate	20	yes	1	3.70	2.72	70	+ +	-
11	78	M	head	adenocarcinoma	CBD	T2N0	0/25	poor	28	yes	2	5.77	2.81	10	+ / + +	-
12	73	M	body-tail	ductal adenocarcinoma	pancreas	T3N1	2/29	intermediate	70	yes	1	3.30	2.10	5	+	-
13	70	M	head	ductal adenocarcinoma	uncertain, likely pancreas	T2N0	0/13	intermediate	28	no	0	n.a	n.a	10	+	-
14	57	M	head	intraoperative biopsy liver: adenocarcinoma	likely pancreas	TxNxM1	unknown	unknown	40	no	0	n.a	n.a	0	-	n.a
15	60	M	body	ductal adenocarcinoma	pancreas	T2N1	1/13	poor	22	yes	1	3.93	2.67	40–50	+ / + +	n.a
16	77	F	tail	ductal adenocarcinoma	pancreas	T2N2	8/18	intermediate	28	no	0	n.a	n.a	0	-	-
17	54	M	head	ductal adenocarcinoma	pancreas	T2N2	11/16	intermediate	38	yes	2	5.66	6.36	70–80	+ +	-

*Abbreviations PSMA* Prostate-specific membrane antigen, *N* Number, *F* Female, *M* Male, *IPMN* Intraductal papillary mucinous neoplasm, *CBD* Common bile duct, *pTNM stage* Pathological tumor-nodal-metastasis stage, *n.a* not applicable, *DM* Diameter, *SUVmax* maximum standardized uptake value, *TBR* Tumor-to-background ratio, *ERG* Erythroblastosis virus E26 transformation specific-related gene

^a^Pathological TNM stage according to the 8th Edition UICC TNM Classification of Malignant Tumours

^b^Positive lymph node ratio, defined as ratio of positive lymph nodes to all lymph nodes

^c^Diameter measured at pathological examination

^d^Radiological PSMA tracer uptake that visually exceeded the blood pool uptake

^e^Visual score in accordance with EANM guidelines, in which 0 is defined as no tracer uptake, 1 as weak uptake, 2 as intermediate uptake, and 3 as strong uptake

### Analysis of ^18^F-PSMA PET/CT scans

**Fig. 1:**
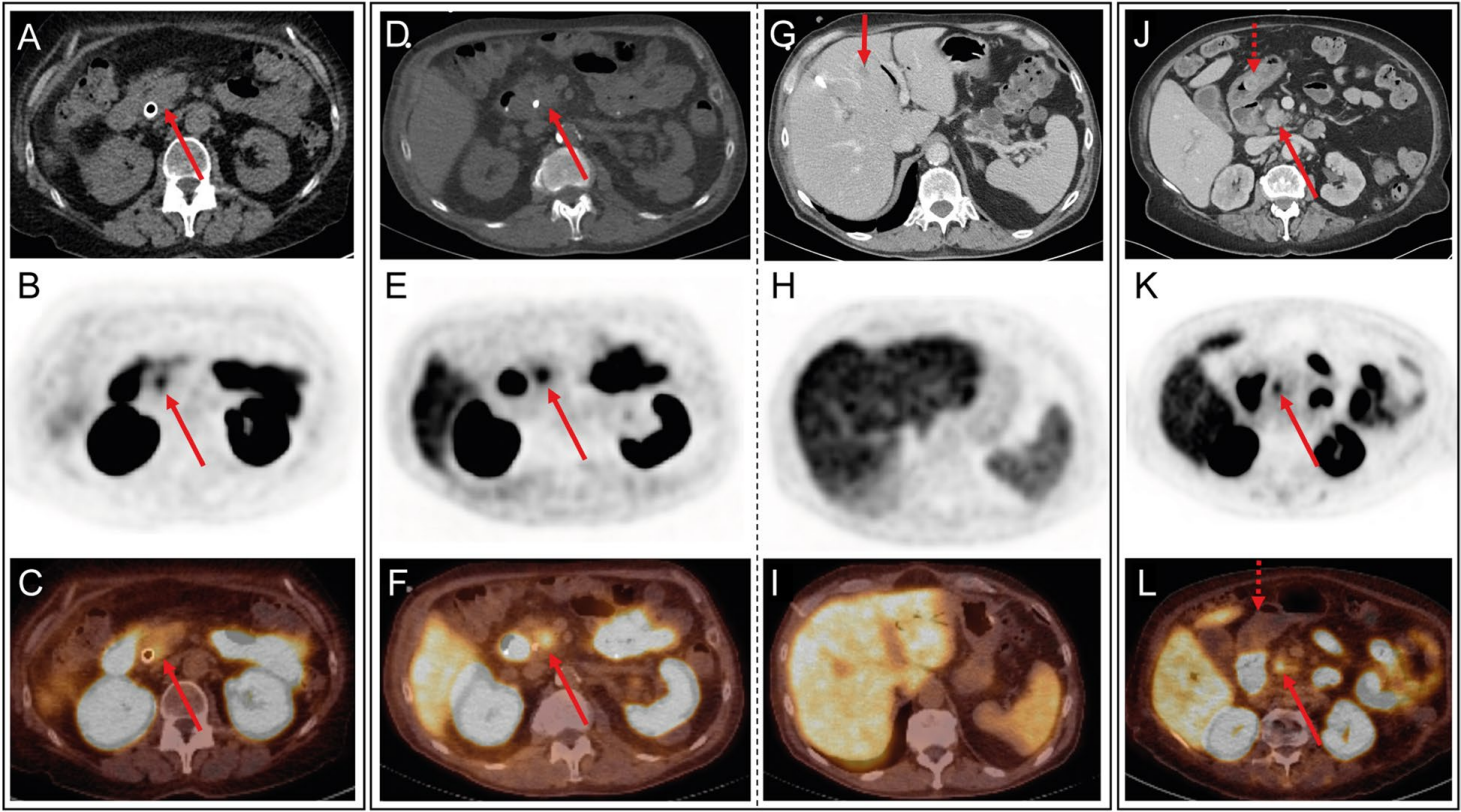
CT, PSMA PET and PSMA PET/CT images of patients with pancreatic ductal adenocarcinoma. **A-C** A 74-year-old female patient with T1cN0 PDAC in the pancreatic head as indicated by the red arrow (patient #8). **D**-**I** A 70-year-old male patient with a suspected pancreatic ductal adenocarcinoma and multiple liver and lung metastases (patient #4). The red arrows show a primary tumor in the uncinate process with PSMA tracer uptake on PET and fused PET/CT images. **G** Another vertical red arrow shows a liver metastasis in segment 4b/5 on CT, which is not avid on (**H**) the ^18^F-PSMA PET and (**I**) fused PET/CT. **J**-**L** A 76-year-old female patient with T1bN0 PDAC (red arrow) and a pT1bN0 primary gastric signet ring cell carcinoma (dashed red arrow; patient #6). Both tumors are visible on (**J**) CT, but only the pancreatic lesion shows PSMA tracer uptake (**K**-**L**). The upper row shows (**A** and **J**) low dose CT or (**D** and **G**) contrast-enhanced CT images, the middle row shows ^18^F-PSMA PET images and the bottom row shows fused ^18^F-PSMA PET/CT images

Out of 17 scans, 13 scans were reported positive for PSMA tracer uptake (see Table 1). Of the 15 resected specimens, 8 were histologically confirmed as PDAC (i.e., primary PDAC and not malignant degenerated IPMN). Out of these 8 PDAC lesions, 7 (88%) demonstrated elevated PSMA uptake (see Fig. 1). The other 6 PSMA-positive lesions included a distal cholangiocarcinoma, an adenocarcinoma of pancreaticobiliary origin, suspected PDAC (i.e., not resected due to metastases), a duodenal adenocarcinoma, an undifferentiated carcinoma, and a non-invasive IPMN. In (semi)quantitative analysis, mean SUV_max_ was 5.1 ± 1.2 across all positive lesions, and 4.8 ± 1.2 for PDAC only. The suspected tumor was detectable (TBR ≥ 2) with a mean TBR of 3.4 ± 1., and 3.4 ± 1.5 for PDAC only. A PDAC lesion with high visual score and TBR is shown in Fig. 1A-C.

Although locoregional lymph node metastases were histologically confirmed in 57% of the resected malignant specimens (Table 1), none of these metastases were PSMA-avid. Furthermore, one patient was diagnosed with 3 lung metastases (long-axis diameters: 3, 5 and 7 mm) and 3 liver metastases (long-axis diameters: 5, 7 and 12 mm), which were visible on diagnostic contrast enhanced CT, but not on ^18^F-PSMA PET (Fig. 1D-I). Another patient did not show any distant metastases on diagnostic CT or PSMA PET/CT, but was diagnosed with a liver metastasis during diagnostic laparoscopy. Two months later, a repeated CT scan demonstrated 3 liver metastases (long-axis diameters: 11, 13 and 17 mm).

Coincidentally, several patients presented with a second primary tumor. A concomitant pT1bN0 primary gastric signet ring cell carcinoma (32 mm) in the gastric antrum was detected on contrast-enhanced CT (Fig. 1J), but did not show tracer uptake on ^18^F-PSMA PET/CT (Fig. 1K-L). Similarly, a cT1aN0 renal cell carcinoma was detected on CT, but was not PSMA-avid. Furthermore, 4 out of 9 male patients showed substantial tracer uptake in the prostate, for which further analysis was recommended: one of these patients deceased due to progressive PDAC before further analysis; another patient entered routine follow-up of prostate-specific antigen (PSA) levels; one patient was treated with bicalutamide hormonal therapy and then entered follow-up; and another patient was finally diagnosed with a cT2/iT3aN0M0 high risk (Gleason 9) prostate carcinoma.

### Immunohistochemical analysis of PSMA protein expression in tumor tissue and relation with PET

**Fig. 2:**
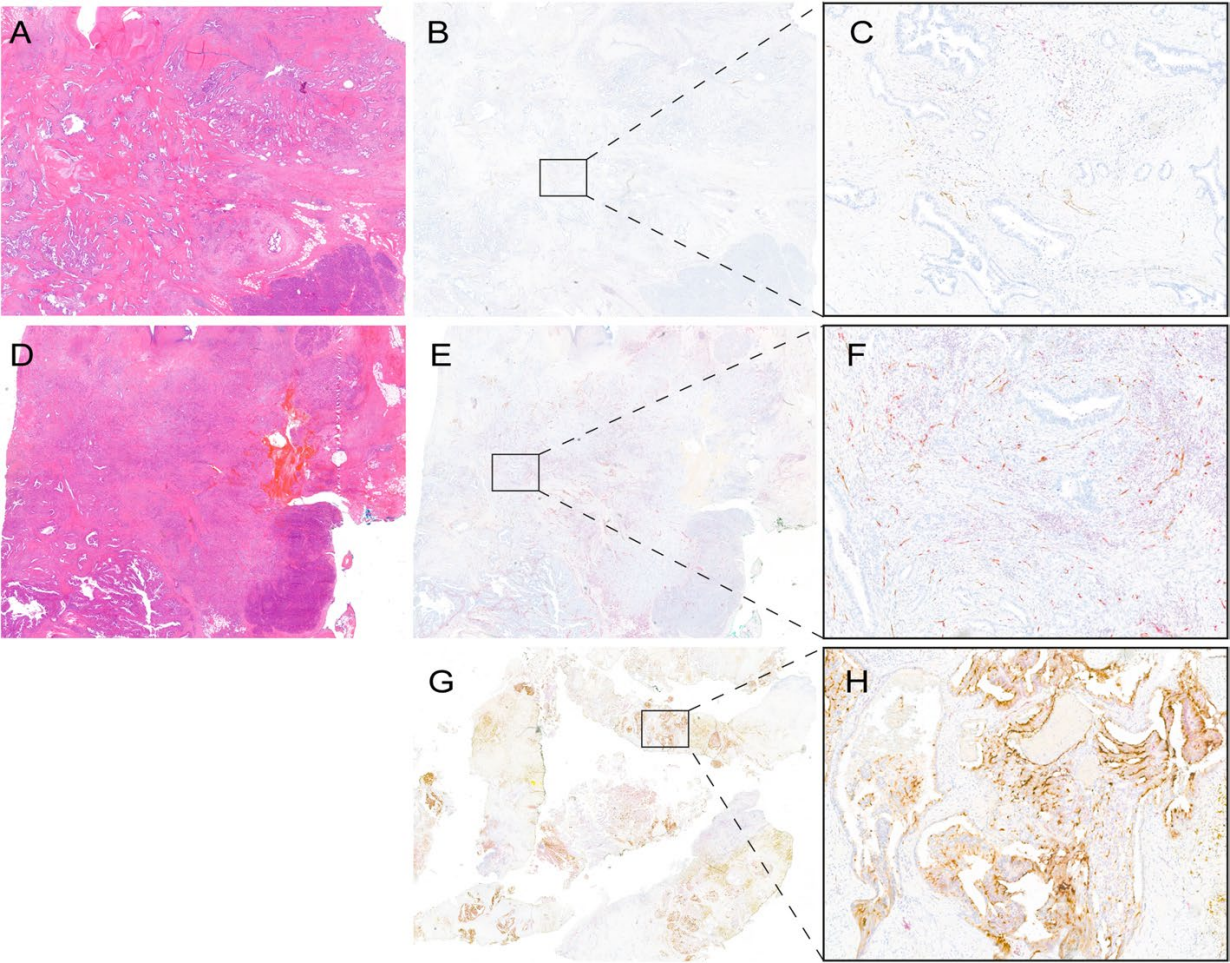
Immunohistochemical staining with Hematoxylin and Eosin staining (left) and PSMA-ERG staining (middle and right). **A-C** A tissue slide of well-differentiated PDAC shows only 10% positive PSMA/ERG staining, but substantial PSMA positive staining in ERG negative structures, as well as negative PSMA staining in pancreatitis (patient 10). **D-F** Tissue of a moderately differentiated PDAC with 70% PSMA/ERG staining and negative PSMA staining in IPMN (patient 6). **G-H** Prostate cancer as positive control

The results of IHC are displayed in Table 1. Out of 17 patients, 15 showed positive vascular staining for PSMA. Mean PSMA/ERG was 31% ± 23, and 39% ± 27 for PDAC only. A tissue slide with moderately differentiated PDAC and a high PSMA/ERG ratio is shown in Fig. 2A-C. Unexpectedly, three patients demonstrated positive PSMA staining in ERG negative structures which morphologically resembled vascular structures. For example, Fig. 2E-F shows a tissue slide of a well-differentiated PDAC with only 10% positive PSMA/ERG, but with many structures that were PSMA-positive while ERG-negative. Tumor-related pancreatitis was reported in four patients and negative for PSMA staining. Two specimens were negative for both PSMA and ERG staining. One was an intraoperative frozen section of a histologically confirmed liver metastasis. The other specimen was from a moderately differentiated PDAC in the pancreatic tail. Interestingly, these two patients did not show any PSMA-avid lesions on ^18^F-PSMA PET/CT, either. In contrast, patient #17 demonstrated a high PSMA/ERG ratio (70–80%), a high SUV_max_ (5.66) and a high TBR (6.36).

**Fig. 3:**
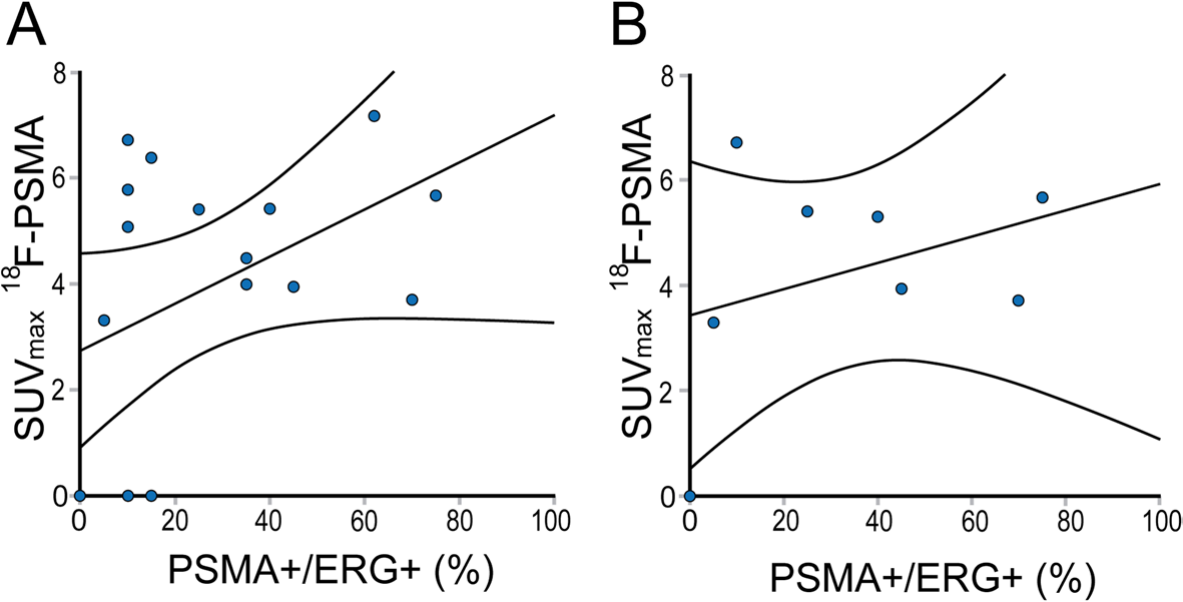
^18^F-PSMA SUV_max_ values with corresponding PSMA + /ERG + percentages for (**A**) all samples and (**B**) PDAC only. Abbreviations: ERG = Ets Related Gene; PSMA = prostate-specific membrane antigen; SUV_max_ = maximum standardized uptake value

Correlation analysis for PSMA/ERG and SUV_max_ is demonstrated in Fig. 3. ^18^F-PSMA SUV_max_ was not statistically significantly correlated to the PSMA/ERG staining ratio (R^2^ 0.186, *p* = 0.084) in the entire cohort, or for PDAC (R^2^ 0.119, *p* = 0.403).

## Discussion

This phase I/II study assessed the feasibility of ^18^F-PSMA PET/CT to detect and stage potentially resectable PDAC. ^18^F-PSMA PET/CT was able to detect most suspected PDAC lesions, but demonstrated low tracer uptake. In this study, none of the malignant locoregional lymph node or distant metastases were PSMA-avid. Moreover, there was no correlation between ^18^F-PSMA PET/CT SUV_max_ and PSMA expression in tissue. PSMA staining was generally positive in tumor-associated neovasculature and negative in areas of tumor-associated pancreatitis.

Two studies previously assessed PSMA uptake in patients with PDAC [30, 31]. Krishnaraju and colleagues assessed the diagnostic accuracy of ^68^ Ga-PSMA PET/CT in patients with malignant (*n* = 19) and benign (*n* = 21) pancreatic disease, and found a 94.7% sensitivity and 90% specificity to detect PDAC [30]. Mean SUV_max_ was 7.4 for malignant and 3.5 for benign lesions. In addition, benign inflammatory diseases showed no tracer uptake. Although this study included a variety of final diagnoses, such as a serous cystadenoma and tuberculosis, results seemed promising. Compared to ^68^ Ga-PSMA tracers, ^18^F-PSMA can be cyclotron-produced and has a longer half-life, which logistically enables a higher production capacity, centralized manufacture and the possibility to distribute to other centers [21, 32]. ^18^F-PSMA is also known to have a shorter positron energy and thus higher spatial resolution, which can contribute to more accurate staging of small metastatic deposits [32]. Another study assessed ^18^F-PSMA PET/CT in 3 patients with PDAC and showed a detectable pancreatic mass in all patients, with a mean TBR of 3.6 ± 1.4 and a mean SUV_max_ of 3.6 ± 1.4 [31]. Although the cohort was small, their mean SUV_max_ is more comparable to our results than the SUV_max_ reported by Krishnaraju et al. [30, 31]. Similar to our findings, no correlation was found between SUV_max_ and PSMA expression in tissue [31]. In addition, both studies did not find or report on any metastatic lesions.

Another objective of this study was to assess the ability of ^18^F-PSMA PET/CT to evaluate detection of PDAC metastases. In literature, two case reports describe PSMA-avid metastases in patients with PDAC and prostate cancer [16, 17]. One case report showed PSMA-avid bone metastases in a patient with rising PSA and history of treated prostate cancer. After initiating treatment, a follow-up ^18^F-PSMA PET/CT showed new and increased bone and liver metastases, while PSA levels were stable. Liver biopsy demonstrated a metastasis from pancreatic acinar cell carcinoma [16]. Another case report described an ^18^F-PSMA PET/CT of a patient with prostate cancer, which demonstrated a PSMA-avid lesion in the pancreas, but also lesions in the lungs, the mediastinum, and PSMA-avid abdominal lymph nodes. The pancreatic lesion was histologically confirmed as PDAC, but the metastases were not histologically assessed [17]. In our study, two patients were diagnosed with metastatic disease in the liver and lungs, but these lesions were not PSMA-avid. Initially, the liver metastases of one of these patients were not detected on diagnostic CT, either. Several factors may contribute to low or negative PSMA signal in metastases, including lower uptake, partial volume effect (PVE), and background signal. PVE is the underestimation of a signal due to limited spatial resolution of a PET and the averaging of signal intensities of different tissue types into a voxel. In other words, a small object causes more 3-dimensional blurring, resulting in larger but dimmer image of that signal [33]. Perhaps, some metastases may be too small to be detected by the PET camera. In this study, distant metastases with diameters ranging from 3 to 12 mm were detected by contrast-enhanced CT, but not by ^18^F-PSMA PET. On the other hand, a primary tumor with a diameter of 14 mm was, in fact, PSMA-avid. Additionally, high physiological PSMA uptake in the liver may hamper detection of liver metastases.

Furthermore, in this study, PSMA protein expression in tumor-associated neovasculature was heterogeneous. Ren and colleagues showed PSMA expression in 100/147 PDAC samples, whereas none of 10 pancreatic intraepithelial neoplasia samples demonstrated PSMA expression [25]. A substantial difference in IHC PSMA expression has also been demonstrated for PDAC, tumor associated pancreatitis and normal pancreatic parenchyma [24]. Another study demonstrated PSMA positive tumor-associated neovasculature in 43 of 81 samples, whereas tumor cells were PSMA positive in only 5% of the samples [26]. Similarly, our study showed that tumor cells did not stain positive for PSMA.

Limitations of this explorative study include a limited sample size and the recruitment of a heterogeneous patient population with several final diagnoses other than PDAC. Nonetheless, this variety of final diagnoses reflects the true patient distribution and is exemplary for the diagnostic challenges of suspected PDAC. Furthermore, patients with neoadjuvant treatment were excluded as the effects of neoadjuvant chemotherapy on PSMA protein expression are unknown. Following several promising case reports, this study is the first phase I/II study to (a) provide evidence for the limited applicability of ^18^F-PSMA PET/CT in patients with suspected PDAC and (b) question whether further studies are warranted.

## Conclusions

This study showed that detection of PDAC using ^18^F-PSMA PET/CT is feasible. However, future application appears to be limited due to low tracer uptake in primary lesions, a lack of specificity to PDAC, high physiological uptake in the duodenum and liver, and no uptake in locoregional or distant metastases in this study. Although ^18^F-PSMA PET/CT may not be useful for accurate diagnosis or staging of PDAC, incidental findings of PSMA-avid lesions in the pancreas on ^18^F-PSMA PET/CT conducted for other indications justify further diagnostic investigation and may potentially result in the diagnosis of PDAC.

## Supplementary Information

Supplementary Material 1.

## Data Availability

No datasets were generated or analysed during the current study.

## Abbreviations

CT: Computed tomography
ERCP: Endoscopic retrograde cholangiopancreatography
ERG: Ets Related Gene
EUS-FNB: Endoscopic ultrasound guided fine-needle biopsy
FDG: Fluorodeoxyglucose
FFPE: Formalin-fixed paraffin-embedded
IHC: Immunohistochemistry
IPMN: Intraductal papillary mucinous neoplasm
PDAC: Pancreatic ductal adenocarcinoma
PET: Positron emission tomography
PSA: Prostate-specific antigen
PSMA: Prostate-specific membrane antigen
SUV: Standardized uptake value
TBR: Tumor-to-background ratio
