# Supplementary material for: 18F−Prostate−Specific Membrane Antigen PET/CT imaging for potentially resectable pancreatic cancer (PANSCAN−2): a phase I/II study
Source: Cancer Imaging. 2025 Jan 14;25:2. doi: 10.1186/s40644-025-00822-y (PMC11734402; doi:10.1186/s40644-025-00822-y)
Supplement: Supplementary file 1 — Supplementary Material 1. [file 40644_2025_822_MOESM1_ESM.docx]

Additional File 1

18F-Prostate-Specific Membrane Antigen PET/CT Imaging for Potentially Resectable Pancreatic Cancer (PANSCAN-2): a Phase I/II study

Jisce R. Puik^1,2,*^, Thomas T. Poels^1,2*^, Gerrit K.J. Hooijer^2,3^, Matthijs C.F. Cysouw^2,4^, Joanne Verheij^2,3^, Johanna W. Wilmink^2,5^, Elisa Giovannetti^2,6^, Geert Kazemier^1,2^, Arantza Farina Sarasqueta^2,3^,

Daniela E. Oprea-Lager^2,4^ & Rutger-Jan Swijnenburg^1,2,7,†^

# Full version of Methods

# Study design

The PANSCAN-2 study primarily aimed to determine whether PDAC can be detected by ^18^F-PMSA PET/CT imaging. The secondary objective was to assess the relation between PSMA tracer signal on ^18^F-PMSA PET/CT and histopathologic PSMA expression. All patients received an ^18^F-PSMA PET/low-dose CT scan prior to pancreatic surgery in addition to standard-of-care diagnostic workup, which included a diagnostic thoracic/abdominal CT within 4 weeks prior to the ^18^F-PSMA PET/CT. ^18^F-PSMA PET/low-dose CT scans were scored visually and (semi)quantitatively. Following surgical resection, paraffin tissue specimens were stained and scored for PSMA expression by an experienced pathologist who was blinded to clinical data.

If no PSMA PET signal was detected in the first five patients, the trial would be stopped early. Study related adverse events and suspected unexpected serious adverse reactions (SUSAR) were to be reported. Adverse events were scored as: mild (discomfort noticed but no disruption of daily routine), moderate (discomfort sufficient to reduce/affect daily activity), or severe (inability to work or perform daily activity). A serious adverse event (SAE) was defined in accordance with the ICH guidelines.

# Patient population

Patients were included if they were of 18 years or older, able to give written informed consent, clinically diagnosed with PDAC and eligible for surgery, and were not to undergo neoadjuvant therapy in concomitant clinical trials. Exclusion criteria consisted of: known hypersensitivity to drugs that resemble ^18^F-PSMA, to any of the excipients or to any component of ^18^F-PSMA; inability to undergo PET/CT scanning, for example due to claustrophobia, weight limits or inability to lay down for 30 minutes; inability to undergo routine magnetic resonance imaging (MRI) or CT scan as part of diagnostic work up; women that were pregnant or lactating. The study aimed to include a total of fifteen consecutive patients with surgical specimens. No sample size calculation was performed due to the exploratory nature of this pilot study.

# ^18^F-PSMA PET/CT Acquisition and Analysis

The investigational radiopharmaceutical is a second generation ^18^F-PSMA-ligand known as ^18^F-DCFPyL, or 2-(3-(1-carboxy-5-[(6-[18F]fluoro-pyridine-3-carbonyl)-amino]- pentyl)-ureido)-pentanedioic acid. ^18^F-PSMA was synthesized under good manufacturing practice (GMP) conditions and with a production license at the on-site cyclotron facilities of Amsterdam UMC (Cyclotron B.V.). All products were packaged, labelled and distributed in accordance with the principles of GMP. Trial medications were stored under 15-25° Celsius and under protection from light exposure.

Patients received ^18^F-PSMA intravenously 2 hours prior to the PET/CT-imaging. ^18^F-PSMA injections contained 300 MBq 18F-PSMA diluted in 0.9% NaCl up to 15mL. After 2 hours, a static ^18^F-PSMA PET/CT was performed from the skull base to mid-thigh using the same PET/CT scanner equipped with time-of-flight (TOF) technology (Philips Healthcare, Best, the Netherlands). PET was combined with low-dose CT (120-140 kV, 40-80 mAs) for anatomic correlation and attenuation correction. When the most recent diagnostic, contrast enhanced CT was conducted more than 4 weeks before the date of surgery, it was repeated for clinical purposes to prevent surgical intervention in advanced disease. Images were reconstructed using point spread function modeling (2 mm, EARL 2 standards).

^18^F-PSMA PET/CT analysis was performed by an experienced nuclear medicine physician (DEOL). Images were both visually and (semi)quantitatively interpreted. In accordance with European Association of Nuclear Medicine (EANM) E-PSMA standardized reporting guidelines v1.0, PSMA intensity was qualitatively scored as: weak (score = 0), defined as uptake higher than the blood pool but less than the liver; moderate (score = 2), defined as uptake more than liver but less than salivary glands; or high (score = 3), defined as higher uptake than salivary glands [1]. A scan was considered ‘positive’, when PSMA expression exceeded blood pool uptake. For the (semi)quantitative analysis, volumes of interest (VOI) were semi-automatically delineated using LIFEx (version 6.30; Inserm, Orsay, France). The blood pool was delineated in the descending aorta using a 3x3 pixel region of interest (ROI). Relative PSMA-ligand concentrations were measured using standardized uptake value (SUV), including maximal SUV (SUVmax), mean SUV (SUVmean) and 3D peak SUV (SUVpeak). For all PSMA-positive lesions, target-to-background ratio (TBR) was calculated as maximum intensity (SUVmax) of the primary tumor divided by SUVmax of the aortic blood pool. Tracer uptake PSMA uptake was considered ‘positive’ when SUVmax ≥ 2.5 and the tumor was considered detectable when TBR was ≥ 2.

# Immunohistochemistry

Formalin-fixed paraffin-embedded tissue were sectioned at 4 μm and dried overnight at 37ºC. Sections were deparaffinized in Xylene and re-hydrated gradually through decreasing concentrations of ethanol. To remove endogenous peroxidase activity, sections were incubated in 0.3% hydrogen peroxidase in methanol for 15 minutes. Antigen retrieval was achieved by 20 minutes of high-pressure cooking in TRIS/EDTA buffer (10 mM Tris Base, 1 mM EDTA solution, pH 9.0 Following antigen retrieval, the slides were incubated for 60 minutes with the PSMA antibody (Clone 3E6, 1:100, DAKO). After washing in PBS-t, the slides were incubated for 30 minutes with a second antibody, Goat anti-Mouse Poly HRP. Bound antibody was detected using Bright DAB+ detection kit (Immunologic, #BS04-110).

Since PSMA is expressed at the membrane, vascular staining was performed using a nuclear antibody: rabbit mAb erythroblastosis virus E26 transformation specific-related gene, known as ERG (Clone EP111, 1:200, Cell Marque Tissue Diagnostics). The slides were incubated for 60 minutes with ERG. Next, slides were incubated for 30 minutes with Goat-anti-Mouse Poly HRP. For PermaRED detections, the slides were incubated with PermaRED Alkaline Phophatase substrate in the dark for 10 minutes (Diagnostic Biosystems). Next, they were counterstained with Hematoxylin (1:5) for 5 minutes. After washing the slides, they were dried completely at 56 ºC degree Celsius to then be mounted.

Histopathological analysis and grading were performed in accordance with the Eight Edition of UICC TNM Classification of Malignant Tumours [2]. PSMA expression patterns were visually assessed by an experienced pathologist who is specialized in pancreaticobiliary pathology. PSMA expression was determined as the ratio positive vessels in relation to ERG positive vessels in the tumor bed. Additionally, staining intensity was determined as weak, moderate or strong. Prostate cancer tissue was used as positive control.

# Statistical Analysis

Statistical analysis was performed using SPSS (version 28; IBM SPSS, INC., Chicago, IL, USA). Data is displayed as mean ± standard deviation. Correlation analysis between SUVmax and percentage of positive PSMA staining over positive ERG staining was performed using logistic regression analysis. A p-value of < 0.05 was considered significant.

**References**

1. Ceci F, Oprea-Lager DE, Emmett L, Adam JA, Bomanji J, Czernin J, et al. E-PSMA: the EANM standardized reporting guidelines v1.0 for PSMA-PET. Eur J Nucl Med Mol Imaging. 2021;48(5):1626-38.

2. Bertero L, Massa F, Metovic J, Zanetti R, Castellano I, Ricardi U, et al. Eighth Edition of the UICC Classification of Malignant Tumours: an overview of the changes in the pathological TNM classification criteria-What has changed and why? Virchows Arch. 2018;472(4):519-31.
